# Supplementary material for: microRNA-22 Inhibition Stimulates Mitochondrial Homeostasis and Intracellular Degradation Pathways to Prevent Muscle Wasting
Source: Int J Mol Sci. 2025 Oct 11;26(20):9900. doi: 10.3390/ijms26209900 (PMC12563987; doi:10.3390/ijms26209900)
Supplement: Supplementary file 1 [file ijms-26-09900-s001.zip › ijms-3859155-supplementary.pdf]

Supplementary figures

Figure S1

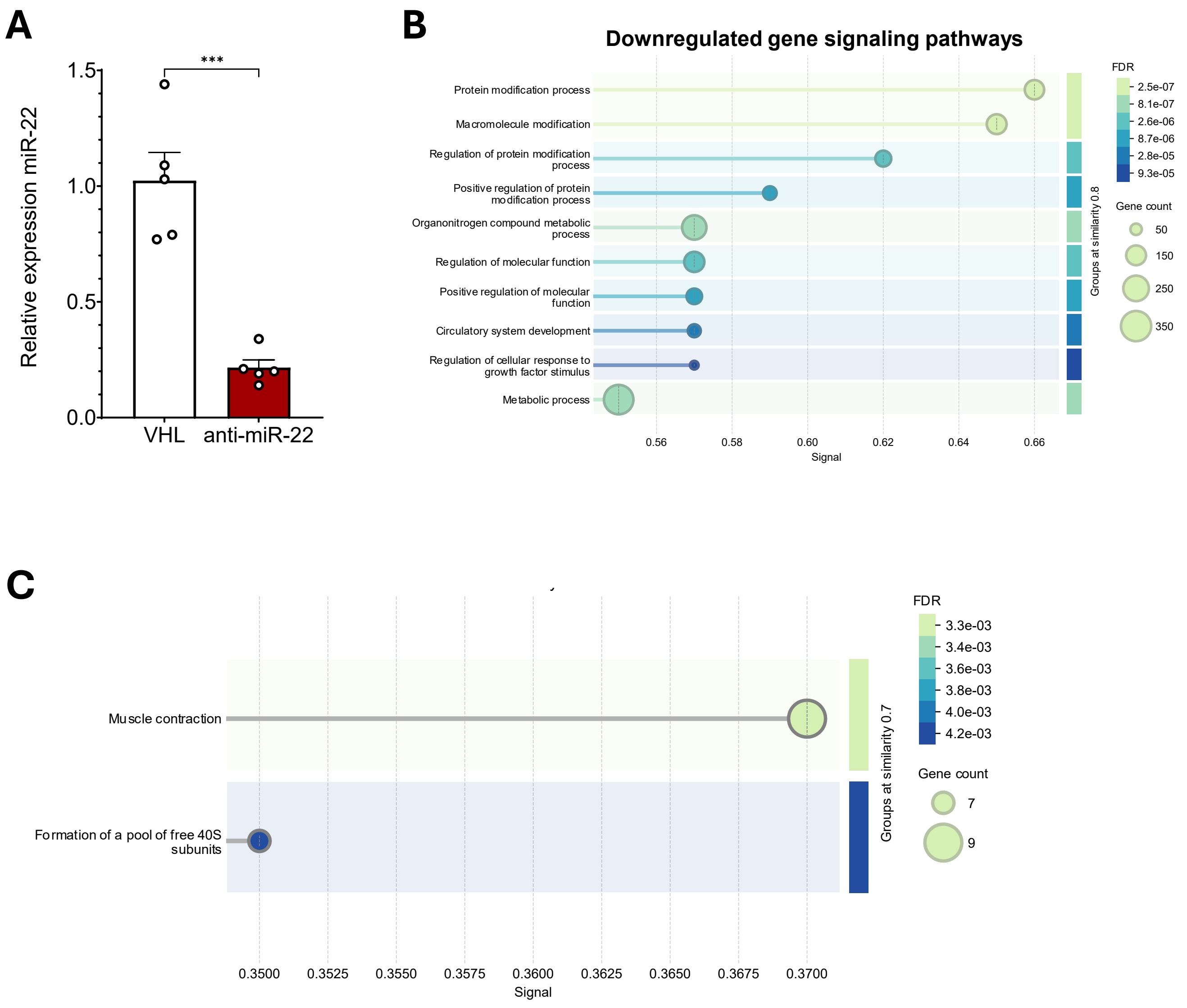

Figure S1 – miR-22 inhibition drives a transcriptional profile supporting muscle contraction in DIO mice

(**A**) miR-22 level in plasma of DIO-WD mice VHL and treated with LNA (n=5). Data are shown as mean ± SEM. (**B**) Gene ontology analysis of downregulated biological processes enriched for differentially expressed genes (DEG) induced by anti-miR-22 ASO in GAS muscle of DIO mice. (**C**) Reactome pathway enrichment of upregulated signaling pathways associated with muscle function induced by anti-miR-22 ASO in GAS muscle compared to HFD control in DIO mice. ***P<0.001 (unpaired t-test).

Figure S2

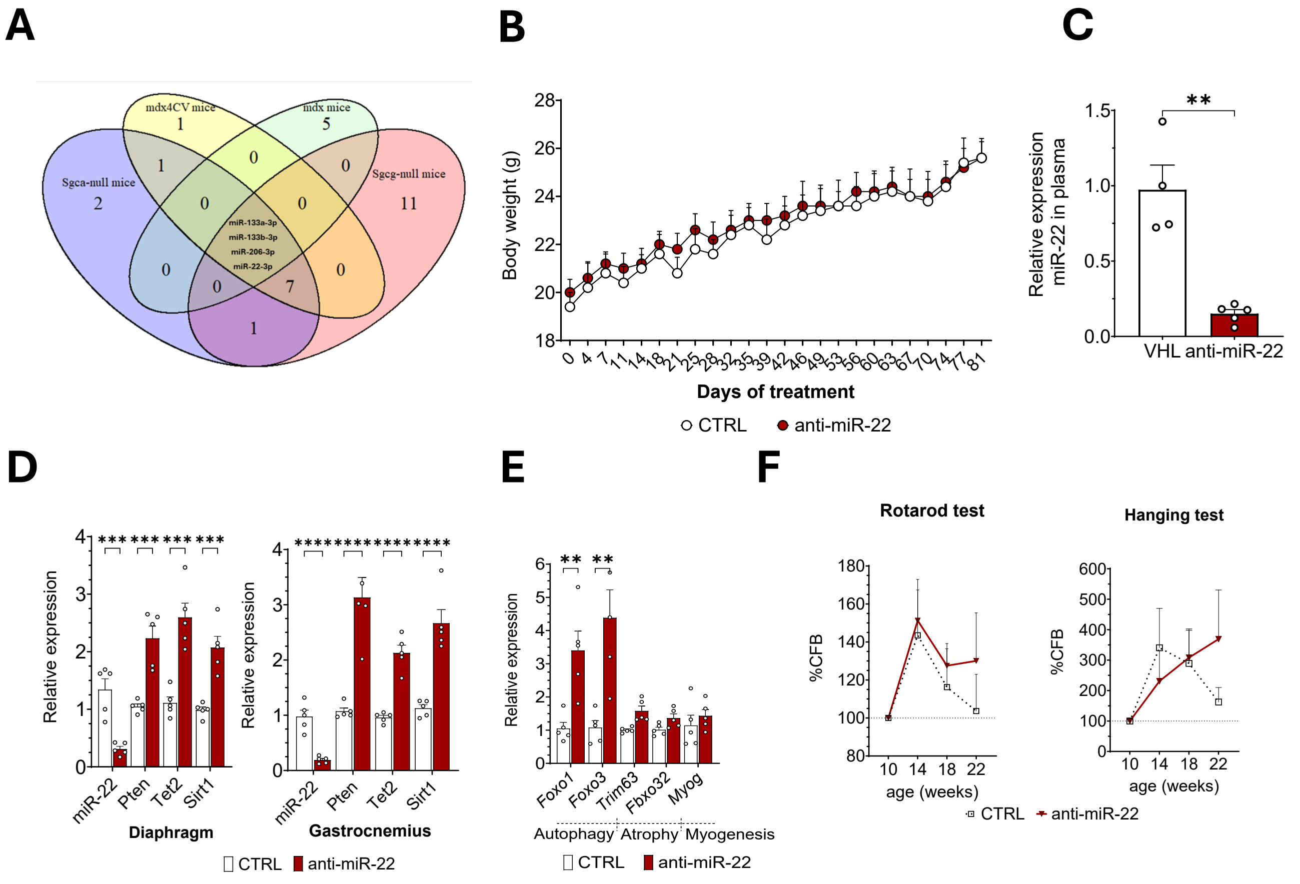

Figure S2 – miR-22 is a targetable biomarker of muscular dystrophy.

(**A**) Venn diagram with upregulated miRNA in the serum of four mouse models of muscular dystrophy (Sgca-null, Sgcg-null, mdx-4CV, mdx mice). Data were extracted and analyzed from published studies aimed at profiling the circulating miRNome in muscular dystrophy (10.1371/journal.pone.0055281; 10.1016/j.omtn.2018.08.005). (**B**) Body weight change of the D2-mdx mice during in the study. (**C**) miR-22 level in plasma of the D2-mdx mice. **(D)** qRT-PCR assessment of miR-22 inhibition and expression analysis of its direct target genes in two striated muscles of D2-mdx mice. (**E**) Assessment of genes related to autophagy induction, muscular atrophy and myogenesis in D2-mdx GAS muscle through qRT-PCR. (**F**) Effect of miR-22 inhibition on locomotor function and fatigue resistance during the study shown as percentage of change from the beginning of the study. The graphs focus on the peak of performance after the first four weeks of the treatment. Data are shown as mean ± SEM. **P<0.01, ***P<0.001, ****P<0.0001 (unpaired t-test).

Table S1

Day	C57BL/6J DIO mice										D2-mdx mice									
	CTRL					anti-miR-22 ASO					CTRL					anti-miR-22 ASO				
	A101	A103	A105	A107	A109	S91	S93	S95	S97	S99	11	12	13	14	15	16	17	18	19	20
0	37.7	46.5	37.9	33.8	45	46	44.8	50.3	49.6	44.7	20	20	18	21	18	18	20	21	21	20
4											21	21	17	23	19	19	21	20	23	20
7	37	47.9	37.4	33.6	45.5	45	44.4	50.1	49.2	44.6	21	21	18	23	21	20	21	21	23	21
11											21	21	18	22	20	19	21	21	23	21
14	37.2	48.3	39	34.4	45.8	45.2	45.1	50.6	49.1	45.5	22	21	18	23	21	20	21	22	22	21
18											22	22	19	24	21	21	21	22	24	22
21	38.6	49.6	40.6	35.6	46.7	46.8	45.7	50.4	48.9	44.7	21	21	19	23	20	20	22	22	24	21
25											23	22	19	24	21	21	23	22	25	22
28	40.5	50.2	41.9	36.3	48.5	47.8	46.2	51	49	45.9	23	21	20	23	21	21	22	22	25	21
32											23	22	20	25	22	20	23	23	25	22
35	40.4	50.3	42.7	35.7	48.4	47.7	46	52	49.7	46.6	24	22	21	25	22	21	23	24	25	22
39											23	22	20	25	21	21	23	24	25	22
42	43.5	50.5	44	37.1	49.6	48	45.7	52.7	50.5	46.3	24	23	21	25	21	21	22	25	25	23
46											24	23	21	26	22	21	22	26	26	23
49	44.8	52.2	44.9	39	51.2	48.4	46.4	52.9	51.2	46.9	25	23	21	26	22	21	23	25	26	23
53											25	23	21	27	22	22	23	25	25	23
56	47.3	52.6	45.4	41.8	51.8	49.6	48.7	55.3	52.7	48.5	25	23	22	26	22	22	23	26	26	24
60											25	24	22	27	22	22	23	25	27	24
63	45.3	51.2	44.9	40.2	51.7	47.1	46.5	52.6	50.4	45.6	25	24	22	27	23	22	24	25	27	24
67											25	23	22	28	22	22	24	25	26	23
70	45.4	52	45.7	41.3	51.9	48.4	47.9	52.4	51.7	46.5	25	23	21	28	22	22	24	25	26	23
74											26	24	22	27	23	22	24	26	27	24
77	47	53.3	45.7	42.5	52.3	48.1	47.9	53.1	52.9	46	26	25	23	29	24	23	25	27	27	24
81											27	25	24	28	24	24	26	27	27	24
84	47.6	53.2	45.8	43.3	52.4	47.5	45.1	51.8	52	45.8										
91	46.3	53.6	45.2	44	52	47	44.4	51.6	52.1	45.2										

Table S1 – Raw body weight data of C57BL/6J and D2-mdx mice (grams).
